# Exploring the Wnt signaling pathway in schizophrenia and bipolar disorder

**DOI:** 10.1038/s41398-018-0102-1

**Published:** 2018-03-06

**Authors:** Eva Z. Hoseth, Florian Krull, Ingrid Dieset, Ragni H. Mørch, Sigrun Hope, Erlend S. Gardsjord, Nils Eiel Steen, Ingrid Melle, Hans-Richard Brattbakk, Vidar M. Steen, Pål Aukrust, Srdjan Djurovic, Ole A. Andreassen, Thor Ueland

**Affiliations:** 1 NORMENT, KG Jebsen Centre for Psychosis Research, Institute of Clinical Medicine, University of Oslo, and Division of Mental Health and Addiction, Oslo University Hospital, Oslo, Norway; 2 Division of Mental Health and Addiction, Møre and Romsdal Hospital Trust, Kristiansund, Norway; 3 Departent of Neurohabilitation, Division of Neurology, Oslo University Hospital, Oslo, Norway; 4 NORMENT, KG Jebsen Centre for Psychosis Research, Department of Clinical Science, University of Bergen, Oslo, Norway; 5 Dr. Einar Martens Research Group for Biological Psychiatry, Center for Medical Genetics and Molecular Medicine, Haukeland University Hospital, Oslo, Norway; 6 Research Institute for Internal Medicine, Oslo University Hospital Rikshospitalet, Oslo, Norway; 7 Section of Clinical Immunology and Infectious Diseases, Oslo University Hospital Rikshospitalet, Oslo, Norway; 8 Instiute of Clinical Medicine, Oslo University Hospital Rikshospitalet, Oslo, Norway; 9 K.G. Jensen Inflammatory Research Center, University of Oslo, Oslo, Norway; 10 Department of Medical Genetics, Oslo University Hospital, Oslo, Norway; 11 NORMENT, KG Jebsen Centre for Psychosis Research, Department of Clinical Science, University of Bergen, Bergen, Norway; 12 K. G. Jebsen Thrombosis Research and Expertise Center, University of Tromsø, Tromsø, Norway

## Abstract

The Wnt signaling pathway plays a crucial role in neurodevelopment and in regulating the function and structure of the adult nervous system. Schizophrenia (SCZ) and bipolar disorder (BD) are severe mental disorders with evidence of subtle neurodevelopmental, structural and functional neuronal abnormalities. We aimed to elucidate the role of aberrant regulation of the Wnt system in these disorders by evaluating plasma levels of secreted Wnt modulators in patients (SCZ = 551 and BD = 246) and healthy controls (HCs = 639) using enzyme immune-assay. We also investigated the expression of 141 Wnt-related genes in whole blood in a subsample (SCZ = 338, BD = 241, and HCs = 263) using microarray analysis. Both SCZ and BD had dysregulated mRNA expression of Wnt-related genes favoring attenuated canonical (beta-catenin-dependent) signaling, and there were also indices of enhanced non-canonical Wnt signaling. In particular, *FZD7*, which may activate all Wnt pathways, but favors non-canonical signaling, and *NFATc3*, a downstream transcription factor and readout of the non-canonical Wnt/Ca^2+^ pathway, were significantly increased in SCZ and BD (*p* < 3 × 10^−4^). Furthermore, patients had lower plasma levels of soluble dickkopf 1 and sclerostin (*p* < 0.01) compared with HC. Our findings suggest that SCZ and BD are characterized by abnormal Wnt gene expression and plasma protein levels, and we propose that drugs targeting the Wnt pathway may have a role in the treatment of severe mental disorders.

## Introduction

Schizophrenia (SCZ) and bipolar disorder (BD) are severe mental illnesses that are leading causes of worldwide disability^1,2^ and are associated with shortened lifespan^3–5^. Early brain morphological findings (i.e., aberrant neural lamination and orientation in the hippocampi), behavioral and cognitive alterations, and nonspecific motor development abnormalities in SCZ were among the first observations that underpinned the neurodevelopmental hypothesis of SCZ^6^. In BD, subtle brain morphological changes, behavioral alterations prior to onset of illness, and attenuations in neurogenesis may indicate disrupted neurodevelopment^7^. Further, in the adult brain, structural alterations, aberrant brain connectivity, and biochemical changes suggest patterns of subtle structural and functional brain abnormalities in both illnesses^8,9^. In addition, some studies have also indicated signs of neurodegenerative processes in SCZ^10^, with weaker evidence in BD^11^.

During neurodevelopment and in the adult brain the Wnt signaling pathway plays a crucial role in neural stem cell proliferation, differentiation and migration, neuroplasticity, and neurogenesis^12,13^. Secreted modulators such as the dickkopf (DKKs) and the secreted frizzled related proteins (sFRPs) regulate both the canonical (β-catenin-dependent) and non-canonical (β-catenin-independent) Wnt signaling, modulating the effects of various Wnt ligands^14^. Initial studies investigating the Wnt signaling pathway in severe mental disorders almost two decades ago focused on the hippocampus, where immunoreactivity of the Wnt ligand Wnt-1 was observed^15^. Aberrant findings in SCZ included reduced β-catenin^16^, increased Wnt-1 expression^15^, and reduced glycogen synthase kinase 3 beta (GSK-3β), a multifaceted kinase in Wnt signaling^17^. Later, large genome-wide association studies identified several polymorphisms in Wnt signaling in SCZ and BD suggesting a strong genetic component^18^. In particular, secreted Wnt antagonists, such as DKK4^19^, sFRP1, and FZD3^20,21^, have been associated with brain volumes^22^ and increased susceptibility to SCZ. In addition, these Wnt antagonists are located in the chromosomal region 8p, which is a genetically implicated region in neuropsychiatric disorders^18^. Furthermore, downstream modulators of β-catenin-dependent signaling have also been linked to increased susceptibility to both SCZ^23,24^ and BD, as well as suicidal behavior^25^ and body composition^26^. The Wnt signaling pathway is also linked to BD through investigations identifying that lithium impairs GSK-3β expression^27^. Although alterations in the Wnt signaling pathway have been evident for the past couple of decades a thorough understanding is still lacking. In particular, whereas most studies on the potential involvement of Wnt signaling in severe mental disorders have focused the canonical pathway, few studies has investigated the role of the non-canonical pathways that may be equally relevant in the pathogenesis of psychiatric disorders^14^. Furthermore, no studies have investigated serum levels of central secreted Wnt antagonists such as DKKs or frizzled related proteins.

In the present study we aimed to further elucidate the aberrations in the Wnt signaling pathway by conducting a pathway analysis on leukocyte mRNA in a large sample of SCZ and BD patients, and healthy controls (HCs) to identify differentially expressed genes as well as comparing circulating levels of secreted Wnt agonists and antagonists. We also aimed to explore associations between the Wnt signaling pathway and the use of medication.

## Subjects and methods

### Study design and ethics

The TOP Study at the NORMENT Centre, Oslo University Hospital, and collaborating Norwegian hospitals^28^ is approved by the Regional Committee for Medical Research Ethics and the Norwegian Data Inspectorate. The biobank is approved by the Norwegian Directorate of Health. All participants provided written informed consent after receiving a complete description of the study.

### Participants

The main inclusion criteria were having a Diagnostic and Statistical Manual of Mental Disorders-IV (DSM-IV) diagnosis of SCZ spectrum disorders or bipolar spectrum disorders, intelligence quotient > 70, and age between 18 and 65 years (for details see ref. ^28^). Healthy volunteers without any history of severe psychiatric disorders (or in any of their first-degree relatives) or substance/alcohol abuse/dependency from the same catchment area were randomly selected from the National Population Registry (www.ssb.no). For the present analyses, patients and controls were not included if they had coexisting autoimmune or inflammatory disease, cancer, ongoing infections, used anti-inflammatory drugs, or had C-reactive protein levels above 20 mg/L. In total, 1625 participants included in the study had available plasma for protein assessment. Of these, 1436 participants (551 SCZ, 246 BD, and 639 HCs) met criteria for inclusion. A subsample of patients and controls (338 SCZ, 241 BD, and 263 HC participants) had available blood samples and passed quality control for microarray analyses.

### Clinical assessments

Diagnosis was obtained using the Structured Clinical Interview for DSM-IV Axis I Disorders^29^. Clinical symptoms were evaluated using the Young Mania Rating Scale^30^, Inventory of Depressive Symptoms^31^, and Positive and Negative Syndrome Scale^32^, while functioning was measured using the Global Assessment of Functioning split version function and symptom scale^33^. The clinical assessment team consisted of psychologists and physicians who were all trained until satisfactory inter-rater reliability was obtained^34^. The use of psychotropic and other medication was also recorded at the time of inclusion. This was based on patient interview and medical records. On the day of blood sampling, the medication list was reconfirmed by asking patients about medication adherence (for details see ref. ^35^), and we also measured serum levels of medication from the same blood sample that was used for gene expression and protein level analyses.

### Plasma protein assessment

We have previously described the blood sampling method^36^. We selected stabile plasma ligands that circulate at a detectable level: DKK1; DKK3; sclerostin (SOST), R-spondin-3 (RSPO3) and sFRP3. We measured their plasma levels in duplicate using commercially available antibodies (R&D Systems, Abingdon, UK) in a 384 format using a combination of a SELMA (Jena, Germany) pipetting robot and a BioTek (Winooski, VT, USA) dispenser/washer. For details see supplementary text 1.

### RNA extraction

Blood samples were collected using Tempus Blood RNA Tubes. Total RNA was extracted with ABI PRISM 6100 Nucleic Acid PrepStation and TEMPUS 12-port RNA Isolation Kit according to the manufacturer’s protocol. High-Capacity cDNA Reverse Transcription Kit was used for reverse transcription of 1 µg RNA.

### Global transcriptomics analyses

We selected 141 Wnt pathway-related genes using the Kyoto Encyclopedia of Genes and Genomes database (http://www.genome.jp/kegg/pathway.html). For each sample, 200 ng of total RNA was biotin-labeled and amplified using the Illumina TotalPrep-96 RNA Amplification Kit (Thermo Fisher, Waltham, MA, USA). Global analysis of gene expression was performed with Illumina HumanHT-12 v4 Bead Chip (Illumina, San Diego, CA, USA) consisting of more than 47 000 probes (i.e., transcripts). For this purpose, 842 samples (263 HCs, 338 SCZ, and 241 BD) passed labeling and scanning. Raw microarray scan files were exported using the Illumina GenomeStudio software and loaded into R for downstream analysis using specific packages provided by BioConductor^37^. Lumi was used to detect outliers^38^. R package (version 3.24.4.) was used to correct for technical batch effects, like RNA extraction batch, RNA extraction method, DNase treatment batch, cRNA labeling batch, and chip hybridization. Further quality control, quantile-normalization, and log2-transformation were done using Limma^39^.

### Statistical analysis

Statistical analyses were performed using the SPSS software package for Windows, version 24.0 for plasma analyses. Data normality was assessed using the Kolmogorov–Smirnov and Shapiro–Wilk tests. We investigated differences in demographic data between groups using the chi-square test for categorical variables, the Kruskal–Wallis test for continuous variables, and the Mann–Whitney *U*-test for post hoc analyses. We used *T*-tests for normally distributed variables, and non-parametric tests (Mann–Whitney *U*-test) for skewed distributions to investigate differences between groups. Correlations were examined by using Spearman’s rank correlation. We controlled for age and sex in linear regression models.

To find associations between expression and the diagnostic group, a linear model was fitted in the R software environment using age, sex, and the expression level of *Bmal1* as covariates. *Bmal1* was included in the analyses to adjust for differences in time of blood sampling and circadian rhythm between patients and controls.

To explore possible associations between medication and the Wnt pathway we first calculated the defined daily dose of psychotropic medications (antipsychotics, mood stabilizers, and antidepressants) according to the guidelines from the World Health Organization Collaborating Center for Drug Statistics Methodology (https://www.whocc.no/atcdd). The defined daily dose is the assumed average maintenance dose per day for a drug used for its main indication in adults. We used serum concentration levels for lithium instead of defined daily dose. We selected significantly regulated Wnt antagonists in plasma (i.e., DKK1 and SOST) or major upstream or downstream differentially expressed genes (i.e., FZD7 or NFATc3). Associations were explored using analysis of covariance where we controlled for age, sex, and other medication groups, and we also investigated whether medication groups would influence these Wnt members (group effects; Supplementary table 2).

We corrected for multiple testing according to the Bonferroni method and alpha was set at *p* < 3 × 10^−4^ for our mRNA analyses differentially expressed genes (investigating 141 genes) and *p* < 0.01 for plasma ligand analyses (adjusting for 5 tests).

### Tissue expression

In addition, we evaluated the tissue expression of FZD7 using the publically available Genotype-Tissue Expression (GTEx) dataset. The GTEx Project was supported by the Common Fund of the Office of the Director of the National Institutes of Health, and by NCI, NHGRI, NHLBI, NIDA, NIMH, and NINDS. The data used for the analyses described in this manuscript were obtained from [https://gtexportal.org/home/gene/FZD7] the GTEx Portal on 06/25/2017 and/or dbGaP accession number phs000424.vN.pN on 06/25/2017. NFATC3 protein expression in the brain was evaluated using The Human Protein Atlas (www.proteinatlas.org)^40^.

## Results

### Demographics and clinical characteristics

The socio-demographic and clinical characteristics of the participants are presented in Table 1. There were significant differences in ethnicity (more Caucasians in the HC group), sex (more males in the SCZ and HC group compared to BD), and age (BD and HC older than SCZ) between the patient groups and HCs. These differences were similar in the plasma (*n* = 1436) and the leukocyte (*n* = 842) cohorts. Patients with SCZ had higher symptom load and lower functioning than BD.

**Table 1:** Demographic and clinical characteristics of participants

Parameters	Plasma (Wnt ligand) cohort				Leukocyte (mRNA) cohort			
	SCZ (*N* = 551)	BD (*N* = 246)	HC (*N* = 639)	Post hoc analysis	SCZ (*N* = 338)	BD (*N* = 241)	HC (*N* = 263)	Post hoc analysis
Male sex, *N* (%)	334 (60.6)	97 (39.4)	364 (57.0)	SCZ > HC > BD	206 (60.9)	94 (39.0)	144 (54.8)	SCZ > HC > BD
Ethnicity (Cauc. %)	444 (80.6)	213 (86.6)	629 (98.4)	HC > BD > SCZ	165 (90.7)	141 (94.6)	208 (100)	HC > BD > SCZ
Medication								
Antipsychotics	510 (84.6)	167 (66.0)	—	SCZ > BD	172 (94.5)	115 (77.2)	—	SCZ > BD
Lithium	12 (2.0)	51 (20.2)	—	BD > SCZ	3 (1.2)	32 (21.6)	—	BD > SCZ
Antidepressants	179 (31.5)	95 (38.8)	—	BD > SCZ	51 (28.0)	58 (38.9)	—	BD > SCZ
Mood stabilizers	56 (9.3)	87 (34.4)	—	BD > SCZ	28 (15.4)	66 (44.3)	—	BD > SCZ
Age (years)	27 (13)	29 (18)	31 (13)	BD, HC > SCZ	25 (11)	36 (20)	36.0 (11)	BD, HC > SCZ
DOI (years)	4 (8)	4 (10)	—	BD > SCZ	5 (9)	5 (13)	—	NS
PANSS total score	62 (22)	44 (13)	—	SCZ > BD	64 (24)	45 (14)	—	SCZ > BD
YMRS total score	3 (9)	2 (5)	—	SCZ > BD	5 (8)	2 (7)	—	SCZ > BD
IDS total score	17 (19)	17 (16)	—	NS	19 (17)	15 (17)	—	NS
GAF-S	40 (15)	57 (16)	—	BD > SCZ	39 (10)	54 (17)	—	BD > SCZ
GAF-F	42 (14)	51 (19)	—	BD > SCZ	40 (11)	50 (17)	—	BD > SCZ

*SCZ* *s*chizophrenia, *BD* bipolar disorder, *HC* healthy controls, *Cauc.* Caucasians, *NS* nonsignificant, *DOI* duration of illness, *PANSS* Positive and Negative Syndrome Scale, *YMRS* Young Mania Rating Scale, *IDS* Inventory of Depressive Symptoms, *GAF-S* Global Assessment of Functioning-Symptom Scale, *GAF-F* Global Assessment of Functioning-Function Scale

Categorical data are given as percent in brackets, while continuous data are given as median with interquartile range. Post hoc analysis is performed using Pearson chi-square for categorical data, and Mann–Whitney *U*-tests for continuous data

### Plasma levels of Wnt modulators

The plasma levels of Wnt ligands in absolute values and group comparisons are summarized in Table 2a. Compared with HCs, patients had significantly lower levels of the Wnt antagonist DKK1 (*p* < 0.01) and SOST (*p* < 0.01 for SCZ and BD), also significant in adjusted analyses (i.e., age and sex). In the SCZ group, the levels of the Wnt antagonist sFRP3 were nominally decreased compared to HC (*p* = 0.04), with no significant differences in plasma ligand levels between SCZ and BD. As for the Wnt agonist RSPO3, we found nominally significant lower levels in SCZ, and no significant differences in BD compared to HC.

**Table 2a:** Differences between patients and controls in Wnt pathway-related ligands

Plasma ligands	M (IQR)			SCZ vs. HC			BD vs. HC			SCZ vs. BD		
	SCZ	BD	HC	*n*	*z*	*t*	*n*	*z*	*t*	*n*	*z*	*t*
DKK1	1.63 (1.76)	1.53 (1.63)	1.99 (2.08)	1245	−4.08***	−4.53***	895	−4.37***	—	856	0.54	0.81
DKK3	27.10 (9.01)	27.80 (8.80)	26.95 (9.02)	1240	−0.64	−0.87	893	1.31	1.07	853	−1.69	−1.03
sFRP3	3.45 (2.34)	3.56 (2.64)	3.88 (2.53)	1245	−2.64**	−2.10*	895	−1.26	−0.71	856	−0.69	−1.23
SOST	134.11 (75.39)	130.97 (68.24)	148.93 (75.39)	1245	−4.13***	−3.86***	895	−3.11**	−2.67**	856	−0.13	
RSPO3	70.9 (49.4)	76.9 (47.6)	76.4 (50.6)	1245	−2.51*	−2.03*	895	−0.80	−0.87	856	−1.07	−0.72

Results are given as *z* from Mann–Whitney *U*-test and *t* from ANCOVA (linear regression analysis) controlling for age and gender. Results are significant after Bonferroni correction if *p* < 0.01, and nominally significant if 0.01 < *p *< 0.05

*M* median, *IQR* interquartile range, *SCZ* schizophrenia, *BD* bipolar disorder, *HC* healthy control, *DKK1* dickkopf 1, *DKK3* dickkopf 3, *sFRP3* secreted frizzled related protein 3, *SOST* sclerostin, *RSPO3* R-spondin-3

*p < 0.05; **p < 0.01; ***p < 0.0003

### Differently expressed genes in SCZ and BD

**Fig. 1:**
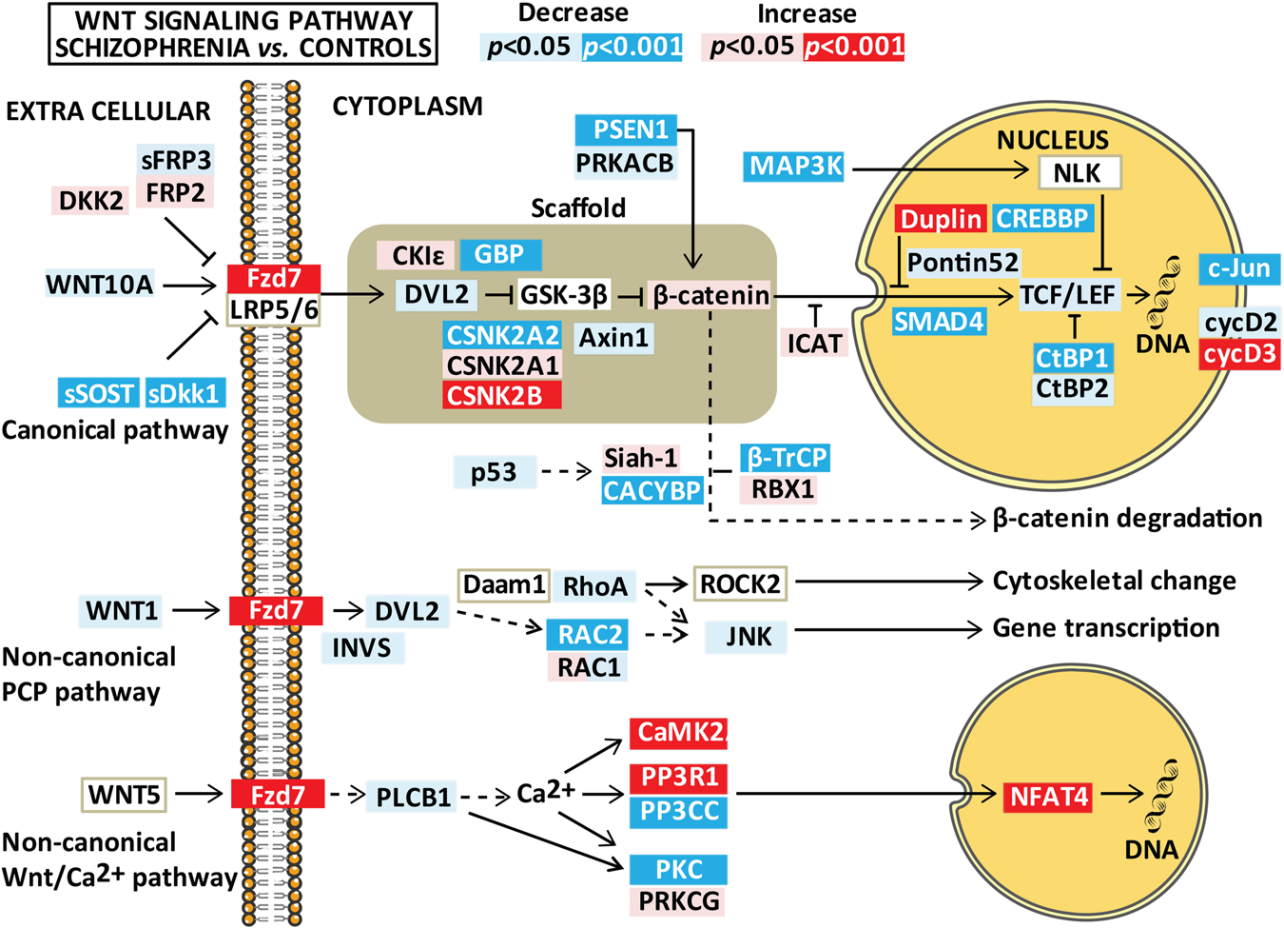
Differences in Wnt pathway gene expression between patients with schizophrenia spectrum disorder and healthy controls after controlling for age, gender, and *Bmal1*. Results are given as *p*-values where significant results (*p*-values adjusted for multiple testing) are indicated in red/dark blue (for increased/decreased mRNA expression) and nominally significant results (0.001 < *p* < 0.05) are shown as pink/light blue (for increased/decreased mRNA expression). The figure summarizes relevant genes for the three Wnt pathways (i.e., canonical pathway, non-canonical planar cell polarity pathway, and the non-canonical Wnt/Ca^2+^ pathway). The figure is based on the Wnt signaling pathway in the KEGG database

**Fig. 2:**
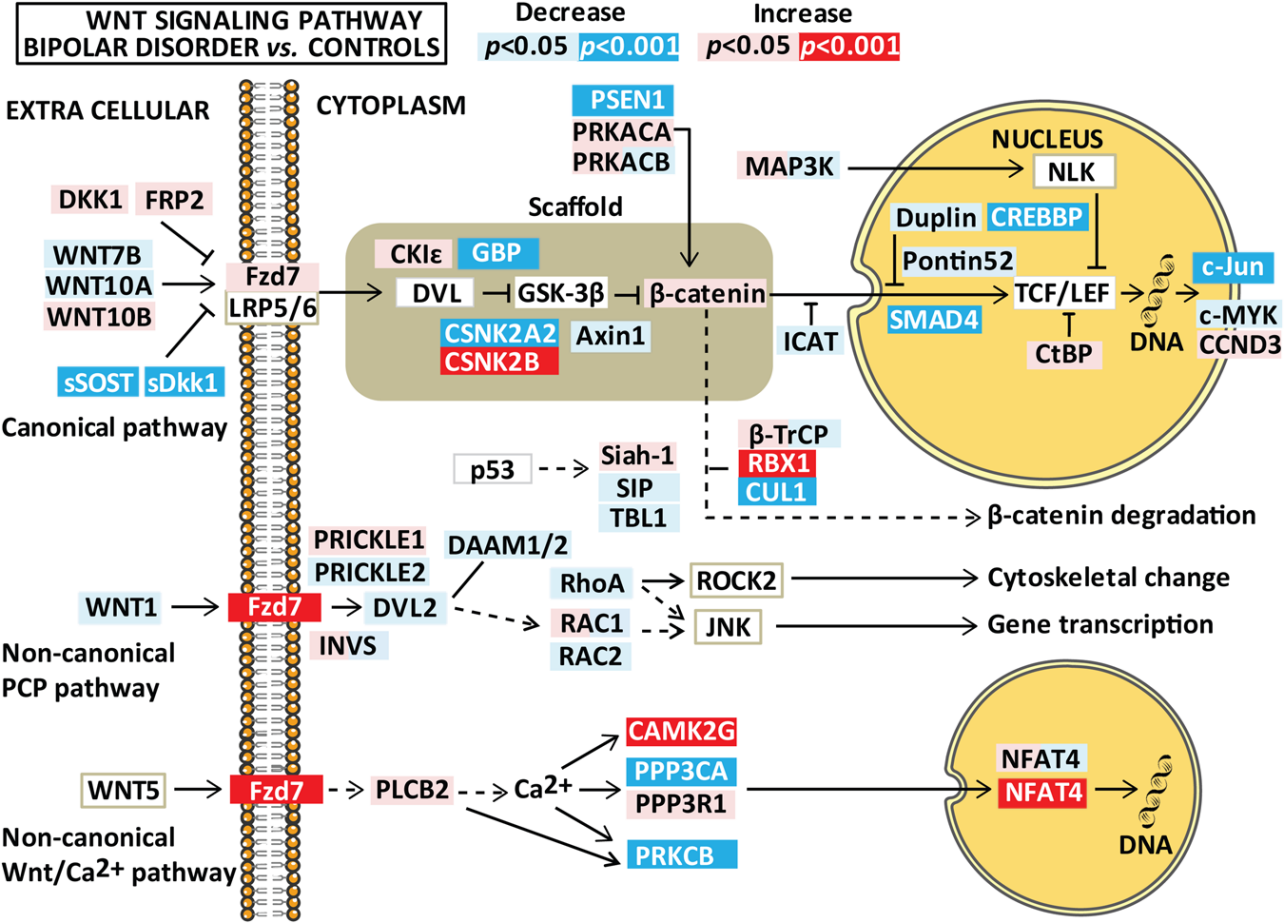
Differences in Wnt pathway gene expression between patients with bipolar spectrum disorder and healthy controls after controlling for age, gender, and *Bmal1*. Results are given as *p*-values where significant results (*p*-values adjusted for multiple testing) are indicated in red/dark blue (for increased/decreased mRNA expression) and nominally significant results (0.001 < *p* < 0.05) are shown as pink/light blue (for increased/decreased mRNA expression). The figure summarizes relevant genes for the three Wnt pathways (i.e., canonical pathway, non-canonical planar cell polarity pathway, and the non-canonical Wnt/Ca^2+^ pathway). The figure is based on the Wnt signaling pathway in the KEGG database

The expression of Wnt pathway genes in whole blood are summarized in Figs. 1 and 2. Genes with significant differential expression (*p* < 3 × 10^−4^) are described in Table 2b, and nominal significant findings (3 × 10^−4^ < *p* < 0.05) in Supplementary table 1. Effect size estimates ranged between |0.03| and |0.15| in significant findings (*p* < 3 × 10^−4^), and between |0.02| and |0.13| in trend level findings (3 × 10^−4^ < *p* < 0.05).

**Table 2b:** Significant differences between patients and controls in Wnt signaling pathway gene mRNA expression after controlling for age and gender in linear models, and correction for multiple testing

	Gene	Protein names	Specificity	SCZ vs. HC	BD vs. HC	SCZ vs. BD
				*B*	*B*	*B*
Wnt canonical pathway	*PSEN1*	Presenilin-1	++	−0.03****	−0.02***	0.01
	*FZD7*	Frizzled-7	++	0.07****	0.07***	0.00
	**β-catenin destruction complex**					
	*FRAT2*	GSK-3-binding protein FRAT2	+++	−0.13****	−0.11***	0.02
	*CSNK2A2*	Casein kinase II subunit alpha	++	−0.05***	−0.05****	−0.01
	*CSNK2B*	Casein kinase II subunit beta	++	0.07****	0.06****	−0.02
	**β-catenin degradation complex**					
	*CACYBP*	Calcyclin-binding protein isoform 2	+++	−0.09****	−0.06**	0.03
	*FBXW11*	F-box/WD repeat-containing protein 11	++	−0.04****	−0.02*	0.02
	*CUL1*	Cullin-1	++	−0.02	−0.06****	0.05**
	*RBX1*	E3 ubiquitin-protein ligase RBX1	++	0.06*	0.13****	−0.07*
	**β-catenin nuclear regulation**					
	*CHD8*	Chromodomain-helicase-DNA-binding protein 8	+++	0.13****	0.09***	−0.04
	*CREBBP*	CREB-binding protein	+	−0.10****	−0.09****	0.01
	*SMAD4*	Mothers against decapentaplegic homolog 4	+	−0.09****	−0.06****	−0.03*
	*CTBP1*	C-terminal binding protein 1	++	−0.03****	−0.01	−0.02*
	*MAP3K7*	Mitogen-activated protein kinase kinase kinase 7	+	−0.03****	−0.03**	0.01
	**Transcription targets**					
	*JUN*	Transcription factor AP-1	+	−0.10****	−0.10****	0.00
	*CCND3*	G1/S-specific cyclin-D3	+	0.11****	0.07**	0.04
Wnt non-canonical PCP pathway	*RAC2*	Ras-related C3 botulinum toxin substrate 2	+	−0.07****	−0.05**	0.03
Wnt non-canonical Ca^++^ pathway	*CAMK2A*	Calcium/calmodulin-dependent protein kinase type II subunit alpha	+	0.06****	0.02	0.04**
	*CAMK2G*	Calcium/calmodulin-dependent protein kinase type II subunit gamma	+	0.01	0.07****	−.05****
	*PPP3R1*	Calcineurin subunit B type 1	+	0.15****	0.10**	0.05
	*PPP3CA*	Serine/threonine-protein phosphatase 2B catalytic subunit alpha isoform	+	−0.02	−0.07****	0.04**
	*PPP3CC*	Serine/threonine-protein phosphatase 2B catalytic subunit gamma isoform	+	−0.06****	−0.03	−0.03
	*PRKCB*	Protein kinase C beta type	+	−0.11****	−0.08****	−0.03
	*NFATC3*	Nuclear factor of activated T cells, cytoplasmic 3	+	0.04****	0.04****	0.00

Gene names are listed according to the HUGO Gene Nomenclature Committee and preferred protein names are given in brackets.

Results are given as effect size estimates from the linear regression analysis after correction for age, sex and BMAL1 expression. Results are significant after Bonferroni correction if *p* < 3 × 10^−4^, and nominally significant if 3 × 10^−4^ < *p* < 0.05.

*SCZ* schizophrenia, *BD* bipolar disorder, *HC* healthy control, *B* unstandardized regression coefficient

*p < 0.05; **p < 0.01; ***p < 0.001; ****p < 3×10^−4^

In general, gene expression in patients compared to HCs was quite similar for SCZ and BD with more modest changes in BD. For example, the Wnt receptor *FZD7* expression was significantly increased in SCZ compared to HC (*p* < 3 × 10^−4^) with similar regression coefficients, but trend level changes in BD (*p* < 0.001). No other frizzled receptors or Wnt ligands were significantly regulated in our patient population.

### Differently expressed genes involved in canonical Wnt signaling

The gamma secretase complex protein PSEN1 was decreased in SCZ compared to HC with a similar trend level decrease in BD. Other differentially expressed genes in the canonical pathway included several members of the β-catenin destruction complex (*FRAT2* decreased in SCZ, *CSNK2A2* decreased in BD, and *CSNK2B* increased in both), degradation complex (*CACYBP* and *FBXW11* decreased in SCZ, and *CUL* decreased and *RBX1* increased in BD), and nuclear regulation of β-catenin (*CHD8* increased in SCZ, *CTBP1* and *MAP3K7* decreased in SCZ, and *CREBBP* and *SMAD4* decreased in both). In addition, the transcriptional target *JUN* was decreased in SCZ and BD while *CCND3* was increased in SCZ. The only significant differentially expressed genes between SCZ and BD were increased *ICAT* and decreased *CAMK2G* mRNA expression in SCZ (Table 2b, Supplementary Figure 1).

### Differently expressed genes involved in non-canonical Wnt signaling in whole blood

The only gene involved in the non-canonical planar cell polarity pathway that was differently expressed was decreased *RAC2* in SCZ compared to HC (Table 2b). For the non-canonical Wnt/Ca^2+^ pathway, a complex expression pattern was observed for genes involved in calcium-dependent cell signaling. First, one of the major isoforms of CaM kinase, *CAMK2A*, and *PPP3R1*, belonging to the regulatory subunit of calcineurin (CaN) were increased in SCZ, and *CAMK2G* in BD. Second, isozymes belonging to the catalytic subunit of CaN and protein kinase C (PKC) were downregulated (*PPP3CA* decreased in BD, *PPP3CC* decreased in SCZ, and *PPP3CB* and *PRKCB* decreased in both). Finally, *NFAT3C* was increased in both SCZ and BD.

### Evaluation of tissue expression of FZD7 and NFATC3 from public databases

Our findings so far suggest that upregulation of the Wnt receptor *FZD7* and *NFAT3C*, an important transcriptional factor of the non-canonical Wnt signaling pathway, may be major characteristics of SCZ and BD. To further elucidate these issues, we investigated tissue expression of these proteins in human brain based on available databases. Evaluation of tissue expression of *FZD7* using the GTEx portal revealed the highest expression of *FZD7* in the cerebellum compared to other tissues (Supplementary Figure 2). Protein expression data for *NFATC3* in the brain are available from the Human Protein Atlas and show medium to high expression in all cell types in the hippocampus, cerebral cortex, caudate, and cerebellum (Supplementary Figure 3).

### Role of medication

We found a positive association between defined daily dose of antipsychotics and plasma levels of DKK1 (*β* = 0.13, *p* < 0.01; Supplementary table 2). However, patients using antipsychotics did not have higher levels of DKK1 compared to patients not using antipsychotics. Patients using antidepressants (*n* = 249) had significantly lower levels of SOST (*t* = −2.91, *p* < 0.01) and increased FZD7 expression (*t* = 2.21, *p* < 0.05).

## Discussion

We show for the first time in a large sample that the mRNA expression of *FZD7* and *NFATC3*, two highly relevant Wnt signaling pathway genes, is significantly increased in patients with severe mental disorders compared to HCs. In addition, our pathway analyses indicate attenuated Wnt canonical pathway in patients vs. controls. Our study is also the first to investigate serum levels of secreted Wnt modulators in SCZ and BD, demonstrating significantly aberrant levels of DKK1 and SOST.

Interpretation of differentially expressed genes from signaling pathways is challenging since most intracellular components are not exclusive, but shared by multiple pathways and possibly more dependent on phosphorylation status than transcription levels. They may however aid in identifying dysregulated elements of a pathway that can be evaluated in detail in following studies. A major finding of our study was increased *FZD7* mRNA levels in SCZ, and to a lesser degree in BD compared to controls. Among the frizzled receptors, FZD7 is unique as it can activate all Wnt pathways with non-canonical signaling being predominant^41^ and also oligomerize with all other FZD receptors and further modulate Wnt signaling^42^. Typical non-canonical Wnt ligands such as WNT5A and WNT11 bind FZD7 and activate the planar cell polarity^43,44^ or the Ca^2+^ pathway^43,45^, shown to be involved in SCZ^46^. Although data on FZD7 signaling in neural tissues is sparse, *FZD7* mRNA is detected in multiple regions in the brain with heavily enriched expression in the cerebellum, which is affected in SCZ^47^. If the dysregulated FZD7 signaling is also present in the neural tissues of patients with psychotic disorders, the promiscuity of FZD7 with regard to interactions with other receptors and multiple Wnt pathways make it an appealing candidate for further studies.

Our second major finding regarding gene expression was increased *NFATC3* mRNA expression in patients. *NFATC3* codes the NFAT4 protein, and data from the Human Protein Atlas shows that NFAT4 protein is strongly expressed in brain tissue. Importantly, a recent genome-wide association study identified *NFAT4* as a susceptibility gene in SCZ^48^. In addition to its role in inflammation and activation of immune cells, which is highly relevant in SCZ and BD, NFAT activation may participate in neuronal apoptosis in the CNS^49^. Specifically, NFAT4 activation has been implicated in neuronal loss in the hippocampus after brain injury^50^, is induced in astroglia^51^ and may promote inflammatory responses^52^. Wu et al.^53^, using an approach similar to ours, recently reported a downregulation of *NFATC3* in a substantially smaller SCZ population. This discrepancy could be related to cellular composition as Wu et al. studied peripheral blood mononuclear cells while our samples contain all leukocyte populations, including a large proportion of neutrophils (~70%), and NFAT is an important inducer of inflammatory responses in neutrophils^54^. Non-canonical Wnt ligands can trigger the Wnt/Ca^2+^ pathway leading to activation of calcium-sensitive enzymes, including CaN, Ca^2+^/calmodulin-regulated kinase II, and PKC, which may activate NFAT signaling^55^. We and others have demonstrated that non-canonical WNT5A stimulation enhanced NFAT activity^56,57^. Based on the complex expression pattern of CaN and PKC transcripts, but increased CAMK2 in SCZ and BD, it is tempting to hypothesize that CAMK2 could activate NFAT as has been demonstrated in lymphocytes^58,59^. Regardless of the mechanisms for upregulation, enhanced NFAT4 activity in the brain and/or immune cells could potentially induce neuro-inflammatory responses in SCZ and BD. Of relevance, both NFAT^60^ and CAMK2^61^ may antagonize the canonical pathway.

In support of a previous study^53^ we found evidence of attenuated canonical Wnt signaling in SCZ and BD. This is suggested by decreased mRNA levels of members belonging to the β-catenin destruction complex (i.e., *CSNK2A2* and *FRAT2*), essential for Wnt/β-catenin signaling^62,63^. Aberrant CK2 signaling in the cortex has been linked to altered neurotransmitter release in SCZ^64^. Decreased *PSEN1* would favor enhanced canonical Wnt signaling, but may also have multiple functions outside of the γ-secretase complex. In addition, regulated levels of components involved in p-53-mediated degradation of β-catenin (e.g., the *CACYBP*, *β-TrCP*, *CUL1*, and *RBX1*) could reflect altered turnover of β-catenin, but are difficult to interpret as multiple non-Wnt substrates are processed by this complex. We identified several differentially expressed genes involved in nuclear transport and regulation of β-catenin, such as increased CHD8, a nuclear protein that inhibits β-catenin signaling^65^, and decreased *MAP3K7*. MAP3K7 is also known as TAK1, and studies in TAK^(−/−)^ mice reveal that low levels may adversely affect cerebellar development and neuro-developmentally regulated behavior^66^. Decreased expression of transcriptional co-activators of canonical Wnt target genes such as *CtBP1* (both co-repressor and co-activator), CREBBP, a protein linked to SCZ susceptibility^67^, and the transforming growth factor transcription factor SMAD4 are also compatible with attenuated canonical signaling. These finding are supported by decreased expression of the Wnt target gene *JUN*, which may further reinforce Wnt signaling^68^ and increased CCND3, which in contrast to cyclin D1/D2, is increased in response to inhibition of canonical Wnt signaling^69,70^.

Interpretation of signaling networks in cross-sectional studies is further complicated by that changes in expression may reflect compensatory feedback mechanisms in response to dysregulated signaling. Multiple families of secreted antagonists or modulators may regulate Wnt signaling^71^ and the decreased circulating levels of DKK1, an antagonist of the canonical Wnt pathway, observed in SCZ and BD could reflect a downregulation of DKK1in an attempt to enhance canonical Wnt signaling. In an analogous fashion, we have shown WNT5A stimulates the release of sFRP3, which antagonizes WNT5A-induced NFAT and ERK activity^57,72^. Alternatively, DKK1 may reflect decreased activity in the Wnt canonical pathway as DKK1 is directly regulated by the β-catenin/TCF complex under physiological circumstances^73^. Low serum levels of DKK1 have been associated with increased risk of somatic diseases, and to predict increased mortality in older patients^74^. SOST is also a potent inhibitor of the Wnt canonical pathway^75^, and low circulating levels are frequently seen in relation to enhanced calcification^76^, which is relevant as calcification of the choroid plexus has been associated with regional brain atrophies and neurodegenerative phenotype in SCZ and BD^77^. Serum levels of SOST have not been investigated in severe mental disorders, but are inversely correlated with vitamin D levels^78^, and low vitamin D associates with symptoms in severe mental disorders^79^.

We investigated whether our findings may be attributed to the use of medication. Due to the naturalistic nature of our study most patients were using a combination of psychotropic medication. We detected a significant dosage-dependent association between antipsychotics and DKK1, however we found no group effects of antipsychotics. SOST levels were lower in the group using antidepressants, which is compatible with an activation in canonical Wnt signaling^80^.

Our study has some limitations that should be considered. First, we did not measure phosphorylation status or protein levels of intracellular components of the Wnt pathways, thus our conclusions rely on gene expression data and plasma protein levels. Second, although a post hoc power evaluation revealed an observed power above 0.90 for most transcripts and circulating proteins, observed power was low (below 0.60), for several low-abundant transcripts. Third, many patients were using a combination of psychotropic medications, which made it impossible to investigate effects of medication in monotherapy. We did, however, control for co-medication in the statistical analyses. Lastly, the cross-sectional design of the study hinders us from making strong claims about causality.

In summary, we provide further evidence of altered Wnt signaling in SCZ and BD in a well-powered sample. In particular, we show that *NFATC3* and *FZD7* mRNA expression is increased in peripheral blood of patients, while they have lower serum levels of DKK1 and SOST compared to HCs. Our findings could suggest that drugs blocking the non-canonical Wnt Ca pathway (e.g., WNT5A antagonists) could have a role in the treatment of severe mental disorders and warrants further investigation.

## Electronic supplementary material

Revised supplemetary file
