# Supplementary material for: Exploring the Wnt signaling pathway in schizophrenia and bipolar disorder
Source: Transl Psychiatry. 2018 Mar 6;8:55. doi: 10.1038/s41398-018-0102-1 (PMC5838215; doi:10.1038/s41398-018-0102-1)
Supplement: Supplementary file 1 — Revised supplemetary file [file 41398_2018_102_MOESM1_ESM.doc]

**Supplementary text 1**

**Detailed description of plasma protein assessment.**

Absorption was read at 450 nm with wavelength correction set to 540 nm using an ELISA plate reader (Bio-Rad, Hercules, CA, USA). Intra-/ inter-assay coefficients of variation were: DKK1 (catalog number DY1906) 3.9%/7.2%, DKK3 (DY1118) 4.5%/6.6%, SOST (DY1406) 4.9%/8.8%, RSPO3 (DY3500) 5.2%/5.9% and sFRP3 (DY192) 3.3%/7.4%. We detected no significant diurnal or postprandial variation for proteins. Diurnal variation (n=13, 24 hours, 4 time-points): mean intra-individual CV was 10.0%, p=0.20 for DKK1; 12.8% , p=0.17 for DKK3; 15.5%, p=0.88 for SOST; 19.5%, p=0.089 for RSPO3 and 10.0%, p=0.89 for sFRP3. Postprandial variation (n=13, fasting vs. non-fasting): mean intra-individual CV: 6.7%, p=0.57 for DKK1; 13.8%, p=0.98 for DKK3; 12.7%, p=0.85 for SOST; 21.6%, p=0.07 for RSPO3 and 7.7%, p=0.50 for sFRP3. Detection limits were ≤ 25 pg/mL for all assays except DKK1 and sFRP3 (<80 pg/mL) as defined as 3xSD of assay buffer (n=10).

| Supplementary Table 1. Nominally significant (3x10^-4^< p <0.05) differences between patients and controls in Wnt signaling pathway gene mRNA expression after controlling for age and gender. | | | | | |
| --- | --- | --- | --- | --- | --- |
|  | Gene symbols (protein names) | specificity | SCZ vs. HC | BD vs. HC | SCZ vs. BD |
|  |  |  | *B* | *B* | *B* |
| *Wnt canonical*  *pathway* | *WNT7B* (protein Wnt-7b) | ++ | -.01 | -.01* | .01 |
|  | *WNT8A* (protein Wnt-8a) | ++ | .00 | -.01 | .01* |
|  | *WNT10A* (protein Wnt-10a) | ++ | -.02** | -.02* | .00 |
|  | *WNT10B* (protein Wnt-10b) | ++ | .00 | .02* | -.02* |
|  | *DKK1* (dickkopf-related protein 1) | +++ | .01 | .01* | .00 |
|  | *DKK2* (dickkopf-related protein 2) | +++ | .02* | .01 | .01 |
|  | *SFRP2* (secreted frizzled-related protein 2) | +++ | .02** | .01 | .02* |
|  | *SFRP5* (secreted frizzled-related protein 5) | +++ | .01 | .02** | -.01 |
|  | *CSNK1E* (casein kinase I isoform epsilon) | ++ | .03** | .01 | -.02 |
|  | *DVL1* (segment polarity protein dishevelled homolog DVL-1) | ++ | .01 | -.01 | .02* |
|  | *DVL1* (segment polarity protein dishevelled homolog DVL-1) | ++ | -.01 | .00 | -.01* |
|  | *DVL2* (segment polarity protein dishevelled homolog DVL-2) | ++ | -.02* | -.02 | .00 |
|  | *CSNK2A1* (casein kinase II subunit alpha) | ++ | -.02** | -.01 | -.01 |
|  | *CSNK2A1* (casein kinase II subunit alpha) | ++ | .02* | .02 | .00 |
|  | *FRAT1* (proto-oncogene FRAT1) |  | -.04** | .01 | -.05** |
|  | *AXIN1* (axin-1) | ++ | -.04* | -.04* | .00 |
|  | *CTNNB1* (catenin beta-1) | ++ | -.02 | .01 | -.03* |
|  | *CTNNB1* (catenin beta-1) | ++ | .01* | .01 | .00 |
|  | *CTNNB1* (catenin beta-1) | ++ | .00 | .01* | -.01 |
|  | *PSEN1* (presenilin-1) | ++ | -.03** | -.03** | .00 |
|  | *PRKACA* (cAMP-dependent protein kinase catalytic subunit alpha) | + | .01 | .02* | .00 |
|  | *PRKACB* (cAMP-dependent protein kinase catalytic subunit beta) | + | -.01* | .00 | -.01 |
|  | *PRKACB* (cAMP-dependent protein kinase catalytic subunit beta) | + | .01 | .02*** | .01 |
|  | *PRKACB* (cAMP-dependent protein kinase catalytic subunit beta) | + | -.03*** | -.03** | .00 |
|  | *BTRC* (F-box/WD repeat-containing protein 1A) | ++ | -.02* | -.02* | .00 |
|  | *FBXW11* (F-box/WD repeat-containing protein 11) | ++ | .02 | .04** | -.02 |
|  | *TP53* (cellular tumor antigen p53) | + | -.02* | -.01 | -.01 |
|  | *SIAH1* (E3 ubiquitin-protein ligase SIAH1) | ++ | .02* | .02* | .00 |
|  | *CACYBP* (calcyclin-binding protein isoform 2) | +++ | -.01* | .00 | -.01 |
|  | *TBL1X* (F-box-like/WD repeat-containing protein TBL1X) | +++ | -.03 | -.05* | .02 |
|  | *RUVBL1* (ruvB-like 1) | +++ | -.03*** | -.04*** | .00 |
|  | *RUVBL1* (ruvB-like 1) | +++ | -.03* | -.01 | -.02 |
|  | *MAP3K7* (mitogen-activated protein kinase kinase kinase 7) | + | .04** | .03* | .01 |
|  | *TCF7* (transcription factor 7) | ++ | -.01* | -.01 | -.01 |
|  | *TCF7* (transcription factor 7) | ++ | -.03** | -.01 | -.01 |
|  | *CTBP1* (C-Terminal Binding Protein 1) | ++ | .01 | .03** | .02 |
|  | *CTBP2* (C-Terminal Binding Protein 2) | ++ | -.03*** | -.01 | -.02** |
|  | *MYC* (myc proto-oncogene protein) | + | -.05 | -.07* | .01 |
|  | *CCND1* (G1/S-specific cyclin-D1) | + | -.01* | -.01 | .00 |
|  | *CCND2* (G1/S-specific cyclin-D2) | + | -.08** | -.03 | -.04 |
| *Wnt non-canonical*  *PCP pathway* | *WNT1* (protein Wnt-1) | ++ | -.02** | -.01* | .00 |
|  | *INVS* (inversin) | +++ | .01 | .01* | .01 |
|  | *INVS* (inversin) | +++ | -.02** | -.01 | -.01 |
|  | *INVS* (inversin) | +++ | -.03** | -.03*** | .01 |
|  | *DAAM1* (disheveled-associated activator of morphogenesis 1) | +++ | .01 | -.02 | .03* |
|  | *DAAM1* (disheveled-associated activator of morphogenesis 1) | +++ | -.03 | -.03* | .01 |
|  | *DAAM2* (disheveled-associated activator of morphogenesis 2) | +++ | -.01 | -.02* | .01 |
|  | *RHOA* (transforming protein RhoA) | + | -.05** | -.04* | -.01 |
|  | *RAC1* (ras-related C3 botulinum toxin substrate 1) | + | .02* | .02* | .00 |
|  | *RAC1* (ras-related C3 botulinum toxin substrate 1) | + | -.04* | -.04* | .00 |
|  | *MAPK9* (mitogen-activated protein kinase 9) | + | -.02* | -.02 | .00 |
|  | *PRICKLE1* (prickle-like protein 1) | +++ | -.02 | -.05*** | .03* |
|  | *PRICKLE2* (prickle-like protein 2) | +++ | .01 | .02* | -.01 |
| *Wnt non-canonical*  *Ca^++^ pathway* | *PLCB1* (1-phosphatidylinositol 4,5-bisphosphate phosphodiesterase beta-1) | + | -.02* | -.02 | .00 |
|  | *PLCB2* (1-phosphatidylinositol 4,5-bisphosphate phosphodiesterase beta-2) | + | -.03 | .06* | -.08*** |
|  | *PLCB4* (1-phosphatidylinositol 4,5-bisphosphate phosphodiesterase beta-4) | + | .01 | .00 | .02* |
|  | *PRKCB* (protein kinase C beta type) | + | -.04* | -.03 | .01 |
|  | *PRKCG* (protein kinase C gamma type) | + | .01* | .01 | .01 |
|  | *NFATC1* (nuclear factor of activated T-cells, cytoplasmic 1) | + | -.01 | .01 | .02** |
|  | *NFATC3* (nuclear factor of activated T-cells, cytoplasmic 3) | + | -.01 | -.02* | -.01 |
|  | *NFATC3* (nuclear factor of activated T-cells, cytoplasmic 3) | + | .03** | .03** | .00 |
| **p* < 0.05 ***p* < 0.01 *** 3x10^-4^ *< p* < 0.001  Specificity: + (unspecific, involved in many pathways), ++ (involved in up to 3 additional pathways *e.g.* Notch, NF-kappa), +++ (exclusively Wnt pathway related gene).  Abbreviations: SCZ=Schizophrenia; BD=Bipolar disorder; HC=Healthy controls; B=Unstandardized regression coefficient.  Gene names are listed according to the HUGO Gene Nomenclature Committee and preferred protein names are given in brackets. | | | | | |

Results are given as effect size estimates from the linear regression analysis after correction for age, sex and *BMAL1* expression.

| Supplementary Table 2. Associations between defined daily dose of medication, plasma protein levels and *FZD7*, *NFATC3* expression. Group effects of medicated vs. non-medicated patients. | | | | | | | | |
| --- | --- | --- | --- | --- | --- | --- | --- | --- |
|  | *n* | | DKK1 | SOST | *n* | | *FZD7* | *NFATC3* |
| *DDD associations*: |  | |  |  |  | |  |  |
| Antipsychotics | 612 | | .13** | -.01 | 298 | | .03 | .05 |
| Lithium | 53 | | -.05 | .06 | 30 | | -.03 | -.06 |
| Mood stabilizers | 144 | | .00 | 0.01 | 100 | | -.01 | -.07 |
| Antidepressants | 210 | | -.08 | -.10 | 120 | | -.08 | -.12 |
| *Group effects*: | *n* (yes) | *n* (no) |  |  | *n* (yes) | *n* (no) |  |  |
| Antipsychotics | 693 | 124 | -.52 | -.69 | 348 | 53 | .03 | -.28 |
| Lithium | 63 | 754 | .72 | -1.02 | 35 | 366 | -.27 | -.95 |
| Mood stabilizers | 146 | 671 | -.20 | .20 | 101 | 300 | 1.67 | .27 |
| Antidepressants | 249 | 568 | .14 | -2.91** | 135 | 266 | 2.21* | -.61 |
| **p* < 0.05 ***p* < 0.01  Abbreviations: DDD = daily defined dose; DKK=dickkopf; SOST= sclerostin.  Gene names are listed according to the HUGO Gene Nomenclature Committee.  We used serum concentration of lithium instead of defined daily dosage (DDD). DDD associations are given as standardized *β* from linear regression analyses after controlling for age, sex and other medication. Group effects of medication are investigated by comparing patients with a psychotropic agent (yes) compared to patients without that psychotropic agent (no) while controlling for age, sex and other psychotropic medication in the analysis of covariance. Group effects are given as *t* from ANCOVA analyses. | | | | | | | | |

Supplementary figure 1. Differences in Wnt pathway mRNA expression between patients with bipolar disorder spectrum disorder and schizophrenia spectrum disorder (after controlling for age, gender and *BMAL1*). The figure is based on the Wnt signaling pathway in the KEGG database
Results are given as p-values.


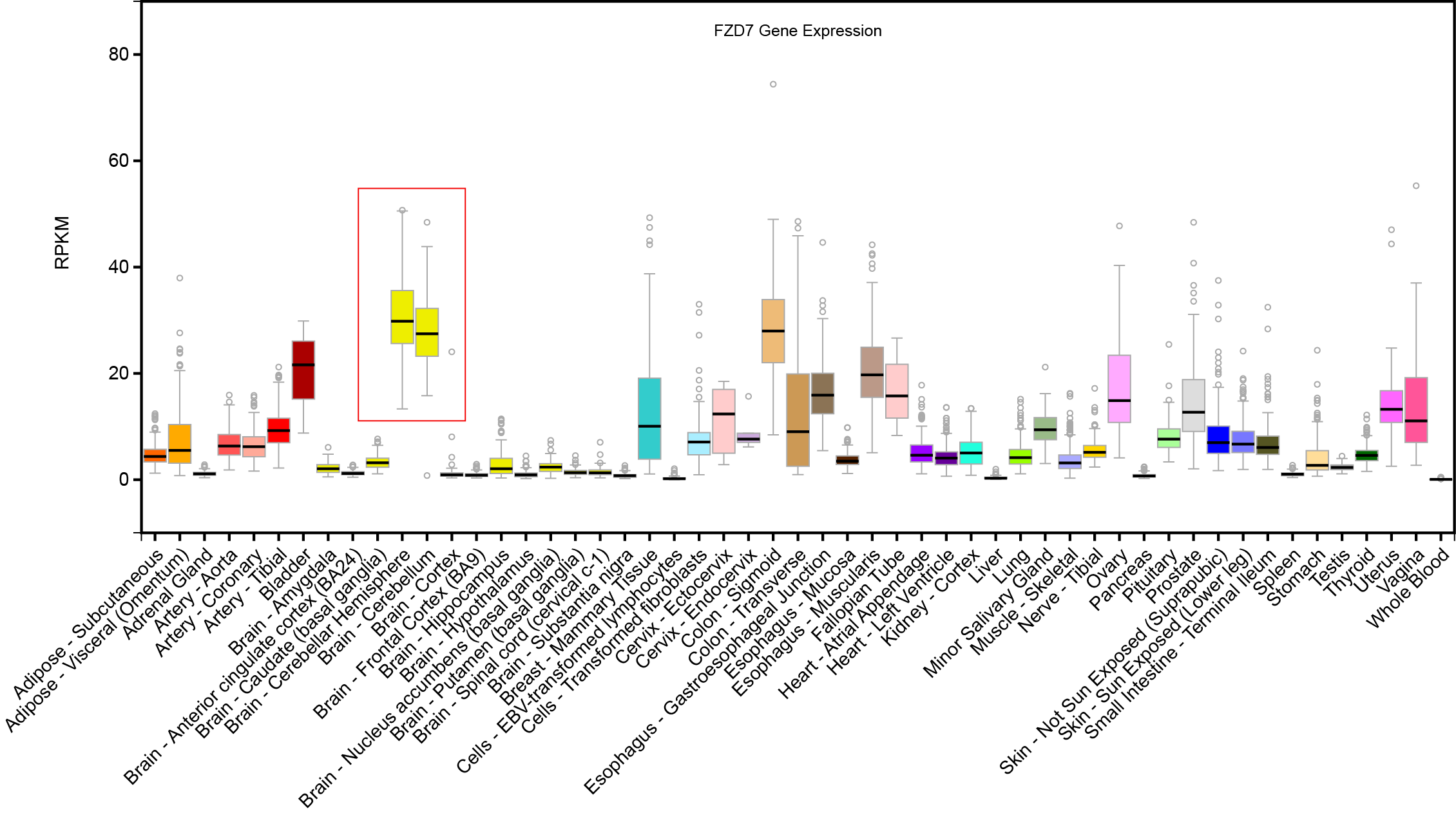


Supplementary Figure 2. Gene expression for *FZD7*. Data source: GTEx Analysis Release V6p (dbGaP Accesion phs000424.v6.p1). Expression values are shown in RPKM (Reads Per Kiolbase of transcript per Million mapped reads), calculated from a gene model with isoforms collapsed to a single gene. Box plots are shown as median and 25^th^ and 75^th^ percentiles; points are displayed as outliers if they are above or below 1.5 ties the interquartile range


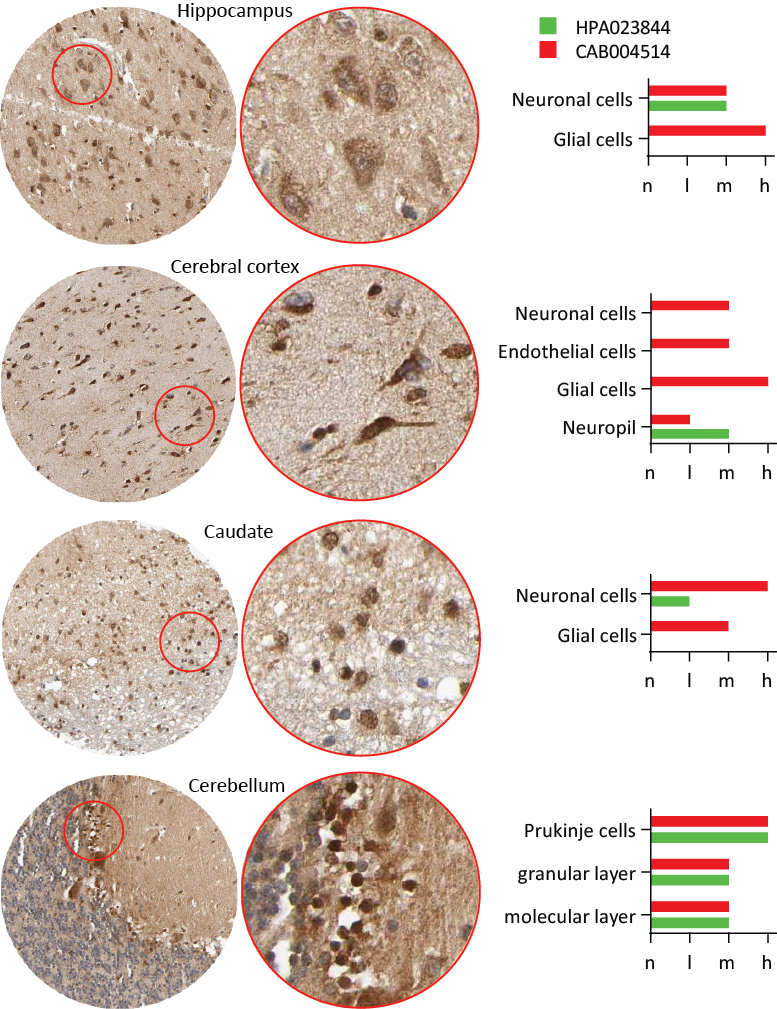


Supplementary Figure 3. Protein expression for *NFATC3* in brain tissues. Data source: The Human Protein Atlas (<http://www.proteinatlas.org/ENSG00000072736-NFATC3/tissue/primary+data>)
